# BeiDou Geostationary Satellite Code Bias Modeling Using Fengyun-3C Onboard Measurements

**DOI:** 10.3390/s17112460

**Published:** 2017-10-27

**Authors:** Kecai Jiang, Min Li, Qile Zhao, Wenwen Li, Xiang Guo

**Affiliations:** 1 GNSS Research Center, Wuhan University, 129 Luoyu Road, Wuhan 430079, China; kc.jiang@whu.edu.cn (K.J.); cheeselee@whu.edu.cn (W.L.); xiangguo@whu.edu.cn (X.G.); 2 Collaborative Innovation Center of Geospatial Technology, 129 Luoyu Road, Wuhan 430079, China

**Keywords:** BeiDou code biases, Fengyun-3C, onboard BeiDou, single-frequency orbit determination

## Abstract

This study validated and investigated elevation- and frequency-dependent systematic biases observed in ground-based code measurements of the Chinese BeiDou navigation satellite system, using the onboard BeiDou code measurement data from the Chinese meteorological satellite Fengyun-3C. Particularly for geostationary earth orbit satellites, sky-view coverage can be achieved over the entire elevation and azimuth angle ranges with the available onboard tracking data, which is more favorable to modeling code biases. Apart from the BeiDou-satellite-induced biases, the onboard BeiDou code multipath effects also indicate pronounced near-field systematic biases that depend only on signal frequency and the line-of-sight directions. To correct these biases, we developed a proposed code correction model by estimating the BeiDou-satellite-induced biases as linear piece-wise functions in different satellite groups and the near-field systematic biases in a grid approach. To validate the code bias model, we carried out orbit determination using single-frequency BeiDou data with and without code bias corrections applied. Orbit precision statistics indicate that those code biases can seriously degrade single-frequency orbit determination. After the correction model was applied, the orbit position errors, 3D root mean square, were reduced from 150.6 to 56.3 cm.

## 1. Introduction

After the US GPS and the Russian GLONASS, the Chinese BeiDou navigation satellite system (BDS) officially began to provide position, navigation, and timing services in most of the Asia-Pacific area on 27 December 2012 and will offer global services by 2020 [1,2]. Unlike the GPS and GLONASS, which use only medium earth orbit (MEO) satellites, BDS also uses satellites in geostationary earth orbit (GEO) and inclined geostationary orbit (IGSO). Currently, its constellation comprises 14 BDS-2 satellites and 5 new-generation satellites. Equipment supporting BDS has been widely used [2]. In particular, the onboard BDS sensors are also applied to low-earth orbiter (LEO) platforms as a key tracking system for precise orbit determination and occultation missions. For instance, the GNSS Occultation Sounder (GNOS) instrument on the Fengyun-3C satellite is the first BDS/GPS compatible radio occultation sounder in the world, developed by the Center for Space Science and Applied Research, Chinese Academy of Sciences [3,4]. The precise orbits of Fengyun-3C were determined by Zhao et al. [5] using onboard GPS- and BDS-only measurement data. The results show that the 3D root mean square (RMS) of overlapping orbit differences can reach the 2- to 3-cm level for the GPS-only solution, and the 3D RMS of orbit differences between GPS- and BDS-only solutions is approximately 15 cm, which is easily affected by the available tracking BDS data.

However, much of the literature argues that BDS code measurements are affected by severe multipath (MP) effects, which inevitably exist in precise applications. The well-known BDS systematic long-term variation was first investigated by Hauschild et al. [6] in the B1 and B2 signals of the BDS M-1 satellite. Montenbruck et al. [7] analyzed BDS code MP variations for all 3 frequency signals using the Multi-GNSS experiment (MGEX) data, which vary by 0.4 to 0.6 m with changes of satellite elevation angle. To tackle this problem, Wanninger and Beer [8] proposed an elevation- and frequency-dependent linear piece-wise correction model for BDS IGSO and MEO satellites. They found a remarkable improvement in their single-frequency point positioning tests after applying the corrections. The BDS GEO satellites remain stationary at an altitude of approximately 36,000 km directly above the equator between 58° E and 140° E. They show very small elevation variations of several degrees in the view field of ground-fixed stations, so the model proposed by Wanninger and Beer [8] are not applicable to GEO satellites using ground data. Therefore, Lou et al. [9] modeled the biases for GEO satellites based on single-differenced fractional cycle biases, but that requires widespread ground stations and still fits only a limited range of elevation variations. One method to overcome the restriction is to put the receiver on space vehicles; for example, on board LEO satellites. Due to the rapid movement of LEO relative to that of GEO satellites, the geometry variations between them can greatly increase, thus onboard observables can be obtained in wide elevation-angle ranges. Even for IGSO and MEO, better sky-view coverage can be obtained with the available tracking data. Fortunately, the onboard BDS measurements collected by the Fengyun-3C satellite can be used for such investigations.

The aim of this study is to offer further insight into the described systematic biases existing in BDS code measurements using the Fengyun-3C onboard data and to model these biases, particularly for GEO satellites. In this paper, the quality of the onboard data and the code multipath is analyzed and these biases are investigated. Then a set of model corrections is given and applied to orbit determination for Fengyun-3C based on single-frequency BDS data. Finally, we present a discussion of the results from different orbit determination solutions, focusing on the orbit precision improvement after applying the model corrections, to verify their success.

## 2. Fengyun-3C Onboard Data Quality Analysis

The GNOS receiver on the Fengyun-3C satellite is used for the GNSS radio occultation mission of China. The receiver has 3 antennas: a positioning antenna, a rising occultation antenna, and a setting occultation antenna [3,4]. With the background described in Introduction, we confined our study to navigation-related measurements from the positioning antenna, which is a wide-beam, hemispherical coverage, low-gain antenna mounted on the top surface of the Fengyun-3C satellite. The measurement types include L1 C/A-code phase, L1 carrier phase, L2 P-code phase, and L2 carrier phase for GPS, and B1I code phase, B1 carrier phase, B2I code phase, and B2 carrier phase for BDS. The results given in the study are based on a 26-day data set collected from the positioning antenna during DOY 062 to DOY 087 in 2015; the sampling interval is 1 HZ.

### 2.1. Measurement Availability

**Figure 1:**
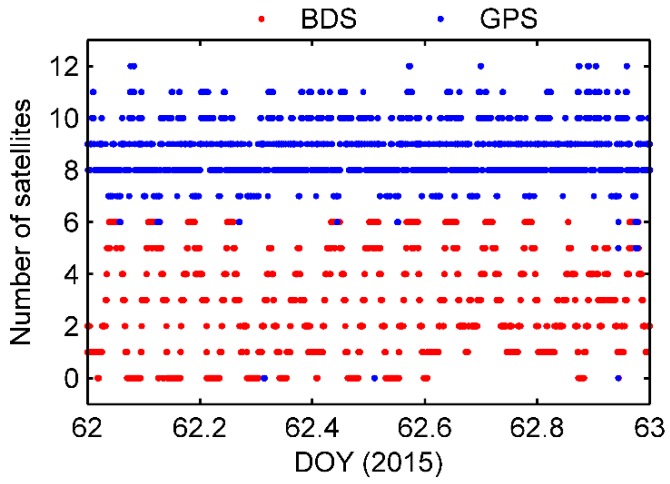
Numbers of BDS and GPS satellites tracked by the onboard GNOS receiver per epoch in DOY 062.

**Figure 2:**
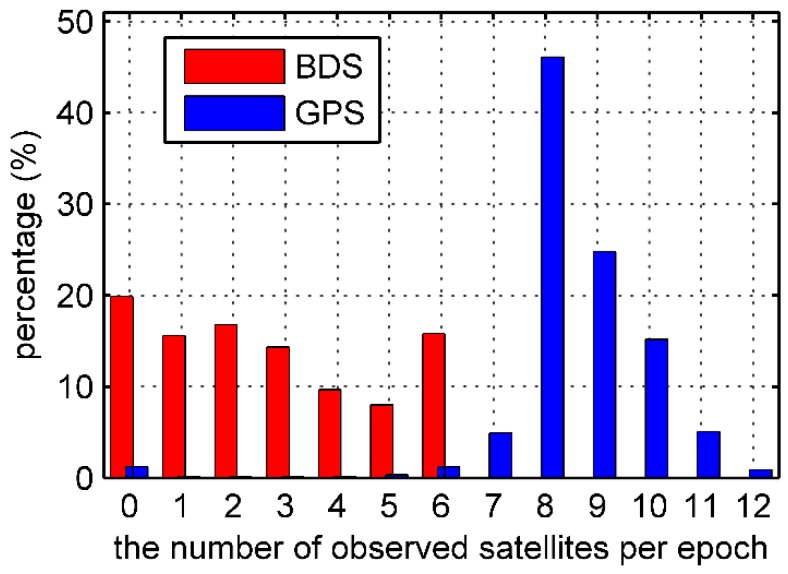
Percentage histogram of the observed GPS and BDS satellites for the 26-day data set.

In the Asia-Pacific regional service of BDS in 2015, there were only 14 operational satellites, including 5 GEOs, 5 IGSOs and 4 MEOs. In addition, the MEO satellite C13 was unavailable in 2015. When orbiting the earth, the Fengyun-3C can receive 4 to 6 BDS signals in most of the Asia-Pacific area because the GNOS instrument is capable of tracking up to 6 BDS satellites from the positioning antenna [3]. However, in other flight regions the tracking number is less than the minimum of 4 satellites required for a kinematic solution. Unfortunately, the GNOS receiver sometimes even fails to track BDS signals. Figure 1 shows the numbers of BDS and GPS satellites that the onboard GNOS receiver tracked per epoch in DOY 062. It is evident that the number of BDS satellites tracked was very unstable due to the invisibility of GEO and IGSO satellites outside the Asia-Pacific region. Compared with the whole GPS constellation, the available BDS data quantity was obviously less. For further comparison, Figure 2 shows the percentage of the observed GPS and BDS satellites for the whole data set. The Fengyun-3C receiver tracked 4 or more BDS satellites in only 33.4% of all epochs, whereas the number of GPS satellites tracked dropped below 4 in just 1.6% of the epochs. The low number of visible BDS satellites is an inevitable problem for the precision orbit determination of the Fengyun-3C satellite, which typically contributes to poor positioning accuracy [10].

### 2.2. BDS Code Multipath

MP effects are caused by non-line-of-sight signal propagation between navigation satellites and the GNOS positioning antenna due to the Fengyun-3C’s assembly parts reflection. In this section, we determine and calculate the MP effects using a linear combination of single-frequency code and dual-frequency carrier-phase observations, namely the MP combination, which can be expressed as [11,12,13]:

(1) $$\begin{array}{cc} MP_{i} & =C_{i}-\frac{f_{i}^{2}+f_{j}^{2}}{f_{i}^{2}-f_{j}^{2}}\lambda_{i}\varphi_{i}+\frac{2f_{j}^{2}}{f_{i}^{2}-f_{j}^{2}}\lambda_{j}\varphi_{j}\\ & +\frac{f_{i}^{2}+f_{j}^{2}}{f_{i}^{2}-f_{j}^{2}}\lambda_{i}N_{i}-\frac{2f_{j}^{2}}{f_{i}^{2}-f_{j}^{2}}\lambda_{j}N_{j}-D_{c} \end{array}$$

where C is the code observables in meters and φ is the carrier-phase observables in cycles; f, λ, and N are frequency, wavelength, and integer ambiguity respectively; the subscripts i and j (i≠j) are used to denote different frequencies; and $D_{C}$ is the constant parts of hardware-induced delays. In theory, this linear combination can eliminate ionospheric and tropospheric delays and all geometric contributions including, for example, clock errors, orbit errors, and the geometry ranges between navigation satellites and receiver, and mainly reflects MP effects and code tracking noise [8,11].

However, the MP combination contains the constant parts of hardware-induced delays and carrier-phase ambiguities. Because these are usually considered as invariant values for each continuous ambiguity block, the practical way to separate them from MP variations is to subtract the mean value of raw MP variations for each block with clean observables and no cycle slips. As a consequence, the MP combination provides only the relative quantity, and the real biases cannot be obtained. For BDS signals, the obtained MP variations can reveal systematic code-carrier divergences, and can be used for code bias modeling.

**Figure 3:**
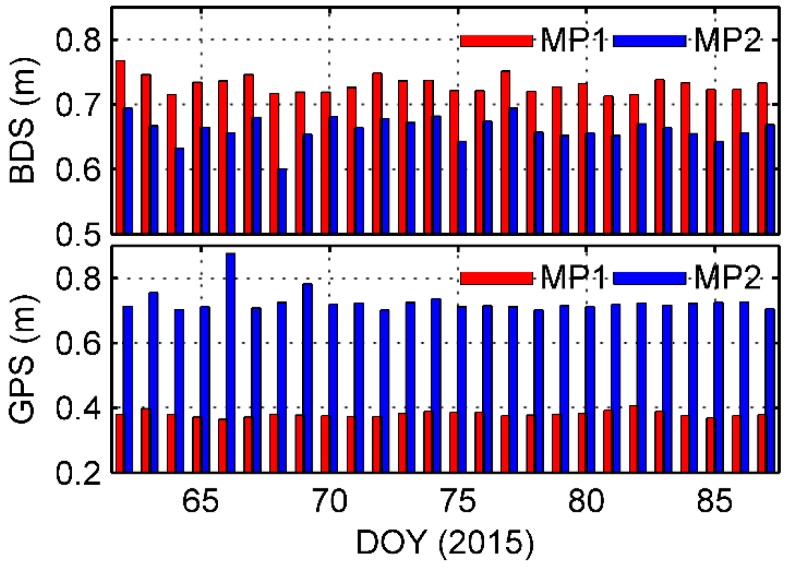
Daily MP1 and MP2 RMSs for onboard BDS and GPS code measurements.

To calculate the MP magnitude of the onboard data set, the daily RMSs of BDS and GPS MP effects were computed for the data above the elevation angle 0°. Figure 3 shows the daily MP1 and MP2 RMSs, and indicates that the variations were very stable. The averaged MP1 and MP2 RMSs are 0.731 m and 0.662 m for BDS, and 0.380 m and 0.724 m for GPS. Compared with GPS MP1, GPS MP2 appears much larger. This is because the SNR of the P2/L2 signals is general poorer than that of C1/L1, with maximum values in our data set being 36.1 and 51.6 dB-Hz respectively.

**Figure 4:**
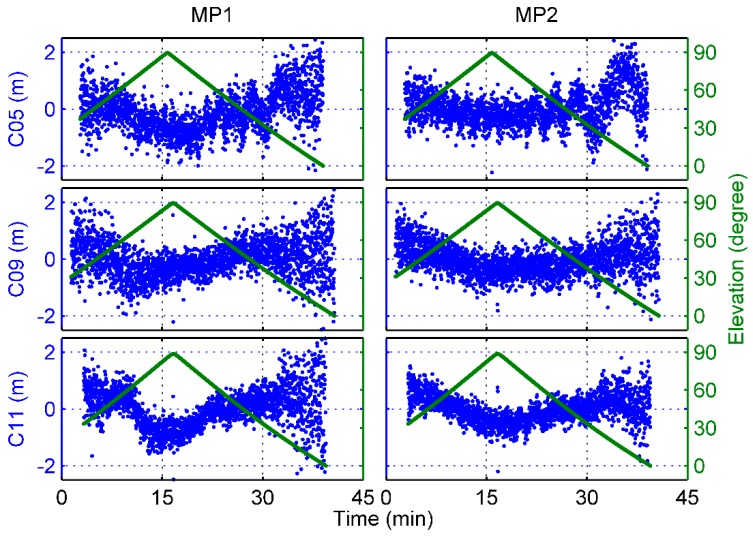
An example of the B1 and B2 frequency MP time series and elevation-angle variations of complete satellite passes as a function of time for different BDS satellite types.

Disclosing additional biases in the MP variations of BDS satellites, Figure 4 shows an example of the B1 and B2 frequency MP time series of 3 complete BDS satellite passes whose maximum elevation angles were close to 90°. The blue dots represent the MP values, and the dark green lines denote the elevation time series. Due to the rapid movement of the Fengyun-3C satellite, the ascending and descending phases of BDS satellites were very rapid and the time interval of a complete satellite pass was only approximately 40 min, which is much smaller than that of ground stations. As illustrated in Figure 4, obvious elevation-dependent biases can be seen, particularly for C11. But it is strange that the MP values do not vary strictly with the elevation values, which is often the case with ground-based BDS MEO data. For C05, there is obviously a periodic signal with unstable wavelength in the time series. The wavelength gradually varies to reach a maximum during the low-elevation range. However, the cause of this phenomenon is still not clear, and we do not discuss it in this paper.

With regard to the onboard data set we used, note that there seems to be an elevation-angle limit for the signals of the ascending navigation satellites. When BDS and GPS satellites appeared in the view field, the elevation angle of observation data was already at an approximately mean value of 30°, as shown in Figure 4. The reason for this phenomenon remains unknown, but it is suspected that some parts of the Fengyun-3C satellite may block the low-elevation signals of the ascending phase of navigation satellites. Fortunately, there was no similar problem for the signals of the descending satellites, and signals at an elevation below 0° could be tracked.

## 3. Results

Using BDS measurements from IGS MGEX stations, Wanninger and Beer [8] modeled the biases in MEO and IGSO satellite passes with a linear piece-wise function of elevation. Because GEO satellites remain nearly stationary as seen from a ground receiver, the similar biases are almost constant and removed when averaging the MP time series of an ambiguity arc. Due to the Fengyun-3C satellite, the problem can be overcome with the onboard BDS data. Good observation coverage can be achieved over the entire elevation and azimuth angle ranges, even for IGSO and MEO satellites.

### 3.1. Inconsistency of Linear Models

**Figure 5:**
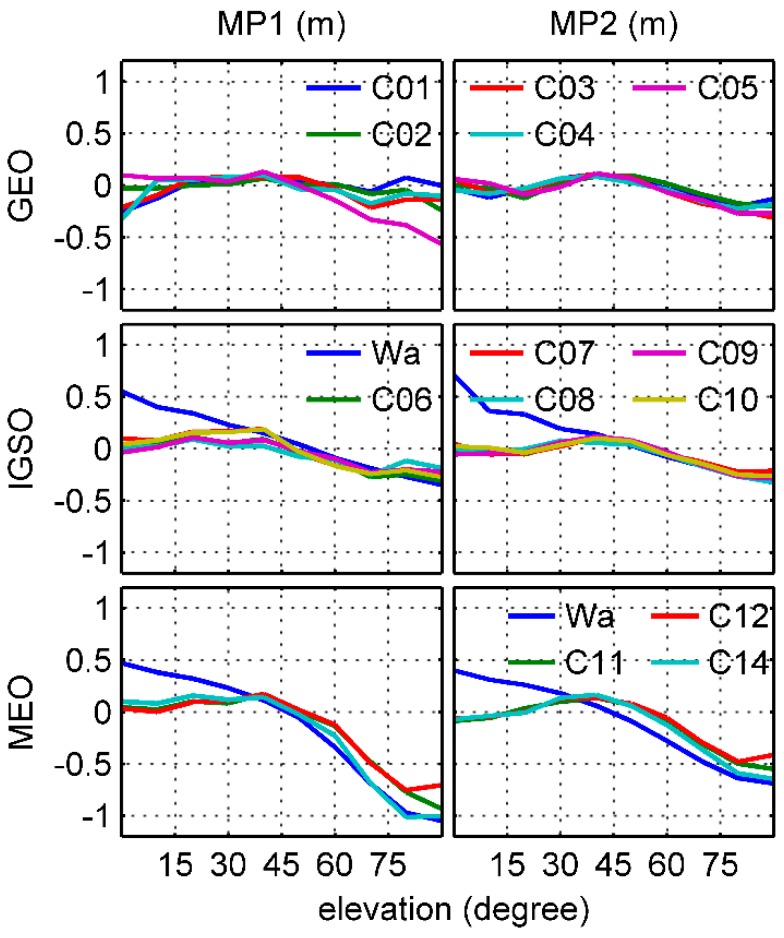
Elevation-dependent MP linear piece-wise models for each BDS satellite from elevation angle 0° to 90° range. For comparison, the 2 blue lines in the IGSO and MEO panels represent Wanninger and Beer’s model.

We estimated the linear model node values using the approach of Wanninger and Beer [8] for individual BDS satellites in a least-squares sense based on the 26-day onboard data set. The results are shown in Figure 5. Similar to Wanninger and Beer [8], groups of satellites with different code bias behaviors are shown. For GEO C01 to C04 satellites, there is no substantial elevation dependence in B1 frequency, but a different behavior can be observed for C05 MP1 under higher elevation angles. About this phenomenon, because we do not have any specific details regarding GEO satellite construction, a new group of MP1 for C05 was taken into account in this study. For IGSO and MEO satellites, there was good agreement with Wanninger and Beer’s model over the high elevation angles for the derived ones, but clear inconsistency can be observed under the lower elevation angles, which were predominantly less than 40°. Zhao et al. [5] thought that this is caused by the Fengyun-3C GNOS receiver, which smooths the BDS code observations when the elevation angle is less than 40°.

However, it needs to be emphasized that because of the loss of the low-elevation observations from the ascending phase of BDS satellites as mentioned above, the inconsistency between the 2 models derives mainly from the descending signals. As seen in Figure 4, the MP values show clear asymmetry with the elevations and the trend of the descending variations is more flat than that of the ascending, which may lead to flat values of the low-elevation nodes shown in Figure 5. A question thus arises as to whether these strange behaviors reflect the actual BDS signal characteristics or a systematic underestimation of the MP variations.

### 3.2. Satellite- and Receiver-Induced Code Biases and Modeling

**Figure 6:**
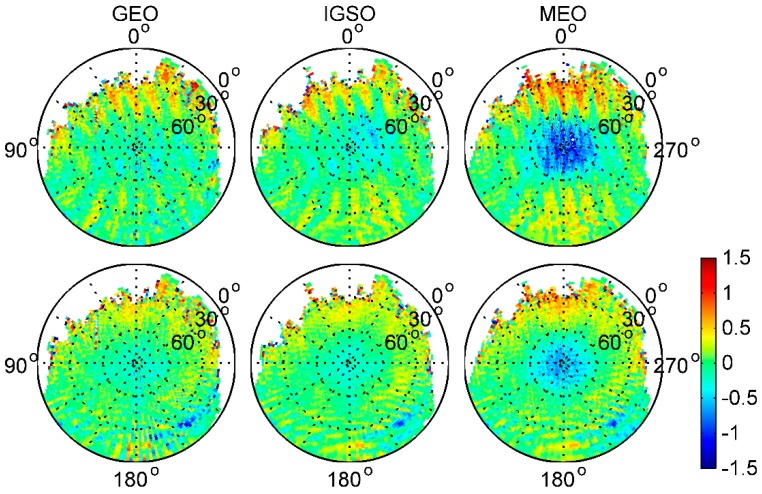
Average 2° × 2° grid map of each BDS satellite type for the BDS B1 (**top**) and B2 (**bottom**) frequencies (unit: m).

**Figure 7:**
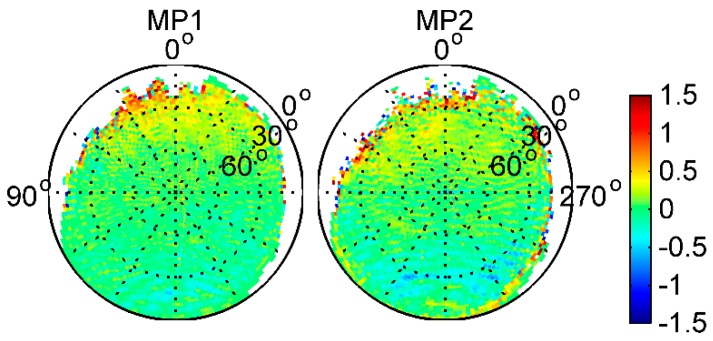
Average 2° × 2° MP grid maps of GPS for L1 (**left**) and L2 (**right**) frequencies (unit: m).

To find out whether there are some other biases in the BDS code measurements, we followed the viewpoint outlined in Montenbruck et al. [14]. MP effects are due to diffuse reflections at the Fengyun-3C body, which results in a static MP pattern that depends only on the line-of-sight direction relative to the GNOS positioning antenna. We thus combined the 26-day MP time series to obtain a good observation sky coverage and estimated the average map on a grid of 2° × 2° resolution in the satellite reference frame for each BDS satellite type. The elevation angles varied from 0° to 90°, as shown in Figure 6, and for comparison, Figure 7 also shows the average MP grid maps of GPS L1 and L2 frequencies. The zero-direction of the zenith antenna pointed along the positive x axis. The satellite reference frame is defined as [5]: the origin of the coordinate system coincides with the Fengyun-3C satellite’s center of mass; the x axis points along the satellite velocity direction, the z axis points toward the center of the Earth, and the y axis completes the right-hand system. In the Figures, the white areas are where there were no available measurements from the signals of the ascending satellites. It is easy to see that the distribution of MPs was not strictly symmetric in azimuth, and some near-field variations with interference fringes are clearly visible. Although it is not clear exactly what caused these interference fringes, a likely cause of the perturbations was cross-talk between the different antenna paths in the GNOS receiver; that is, the signal from one of the occultation antennas leaked into the signal from the positioning antenna. Moreover, when comparing the positive and negative directions of the x axis at the same elevation angles, particularly for elevations below 40°, the biases in the signal of ascending satellites are much larger. In Figure 7, similar perturbations are also obvious for GPS, which is evidence that the near-field effect is not caused by biases in BDS satellites.

**Figure 8:**
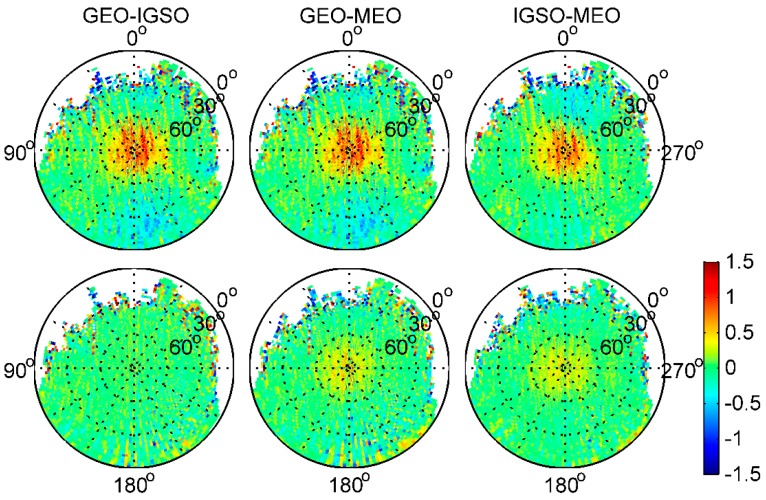
The subtraction between different BDS satellite type grids of B1 (**top**) and B2 (**bottom**) frequencies (unit: m).

Figure 8 shows the subtraction between different BDS satellite type grids in the same BDS frequency, i.e., that IGSO–MEO represents IGSO grid values minus these of MEO in the same frequency. Clearly, the azimuth-dependent variations are obviously eliminated or reduced after subtraction between any 2 BDS satellite type grids. This indicates that there may be some azimuth-dependent systematic biases that were unrelated to BDS satellite types in the onboard BDS measurements. Moreover, according to Wanninger and Beer [8], there should be no substantial azimuth dependence of the BDS satellite-induced biases. Hence, we consider that these biases should have been caused by local MP effects, and not by biases in BDS satellites, which has been demonstrated from the GPS grid maps in Figure 7. This also explains the aforementioned systematic inconsistency under lower elevation angles for IGSO and MEO satellites, when computing the linear piece-wise parameter values regardless of uncalibrated receiver-induced MP effects in the Fengyun-3C environment.

**Figure 9:**
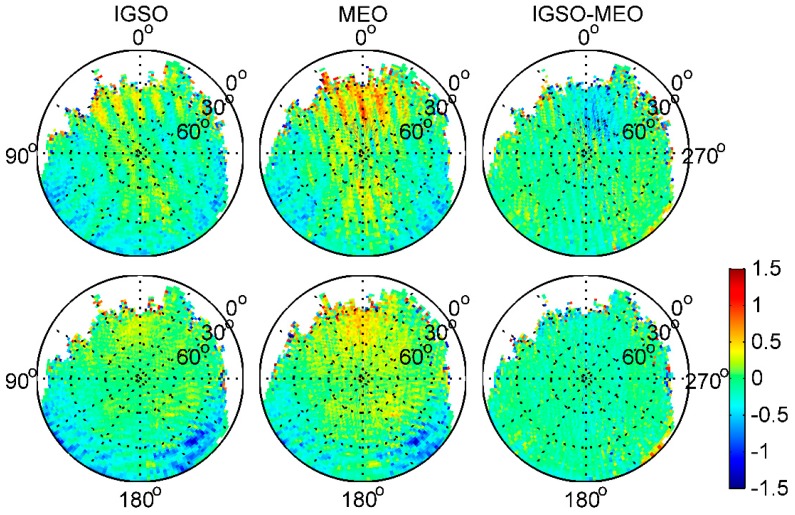
the 2° × 2° resolution grid of the static receiver-induced MP pattern in the Fengyun-3C environment for BDS B1 (**top**) and B2 (**bottom**) frequencies when correcting satellite-induced biases using Wanninger and Beer’s values (unit: m)

To further investigate the static receiver-induced MP pattern in the Fengyun-3C environment, we adopted the model correction values given by Wanninger and Beer [8] to correct the BDS code biases in IGSO and MEO code measurements. Afterward, the sky-view grid maps of the corrected IGSO and MEO MP variations for B1 and B2 frequencies were derived, which were believed to be receiver-induced MP effects; these results are shown in Figure 9. In the IGSO and MEO panels of Figure 9, a decreasing trend of receiver-induced MP effects from the signal ascending direction to the signal descending direction can be observed and is very similar to GPS in Figure 7; it varies by more than 1 m. After subtracting between IGSO and MEO, the trend is significantly reduced. However, some small trend components are still visible. This may be caused by the relatively larger local MP noise or the inaccuracy of the BDS code bias corrections. Based on the analysis, it is quite certain that the static near-field MP pattern exhibits only a frequency-dependent characteristic and is independent on the BDS satellite type.

Therefore, when computing the precise values of elevation-dependent BDS-satellite-induced biases using Fengyun-3C onboard measurements, removal of local MP effects must be considered. Supposing that each of the grid elements consists of 4 node points (A, B, C, and D) and the elevation and azimuth angles of these points are ($e_{1}$, $a_{1}$), ($e_{1}$, $a_{1}$+2°), ($e_{1}$+2°, $a_{1}$+2°), and ($e_{1}$+2°, $a_{1}$) respectively, the MP observables are expressed as

(2) $$\begin{array}{l} MP_{i}^{s}(e,a)=(1-\alpha )(1-\beta )P_{i,A}+\alpha (1-\beta )P_{i,B}\\ \;+\alpha \beta P_{i,C}+(1-\alpha )\beta P_{i,D}-b_{i}^{s}(e)+\varepsilon_{i}\\ \alpha =\frac{\left(a-a_{1}\right)}{2};\;\beta =\frac{\left(e-e_{1}\right)}{2}\\ a_{1}=2[\frac{a}{2}];\;e_{1}=2[\frac{e}{2}] \end{array}$$

with

(3) $$b_{i}^{s}(e)=b_{i,k}^{s}+\frac{e-e_{k}}{e_{k+1}-e_{k}}(b_{i,k+1}^{s}-b_{i,k}^{s})$$

where subscript i and superscript s are used to denote the different frequencies and groups of BDS satellites respectively, P is the receiver-induced MP value of each grid point, ε is the noise, [•] stands for the down rounding operation, $b_{i}^{s}(e)$ is biases in BDS satellites and can be expressed with the linear piece-wise model, and $b_{i,k}^{s}$, $b_{i,k+1}^{s}$ are the bias values of two adjacent nodes. Hence, using Equations (2) and (3) as the observation equation in a least-squares sense, we again estimated 7 groups of elevation-dependent linear piece-wise node values $b_{i,k}^{s}$, including GEO C01 to C04 B1, GEO C05 B1, GEO B2, IGSO B1 and B2, and MEO B1 and B2, together with 2 groups of the receiver-induced MP grid values P for B1 and B2 based on the whole data set. Because of a different behavior observed in C05 MP1 variations as above, a new group was taken into account in this process.

**Figure 10:**
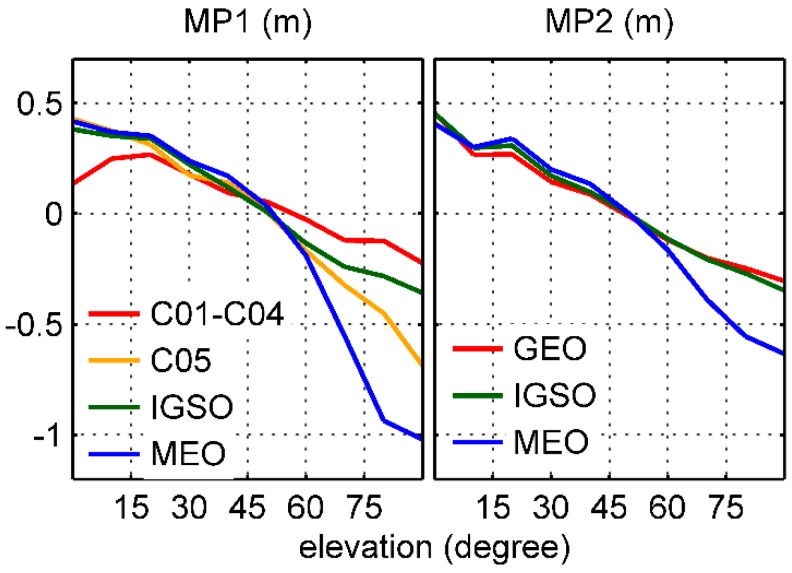
The final derived elevation-dependent MP model.

Figure 10 shows the final elevation-dependent linear piece-wise models which can be expressed as Equation (3) using derived node values $b_{i,k}^{s}$ from 0° to 90° for each group, and Table 1 gives the linear piece-wise model corrections for BDS-satellite-induced biases. Because the distribution of the derived receiver-induced MP grid values P is similar to that in Figure 9, we do not display it in figures. In contrast to Figure 5, clear elevation-dependent variations can be observed in both BDS GEO B1 and B2 frequencies. However, a different behavior did exist in C05 B1 relative to others, and its amplitude was much larger than that of the C01 to C04 group. Comparing different groups, the 2 lines of GEO and IGSO B2 almost overlap together, so they should be placed in the same category. It is also interesting that when the elevation angle was below 60°, the difference between groups in the same frequency was less than 5 cm, except for the C01 to C04 B1 group.

**Figure 11:**
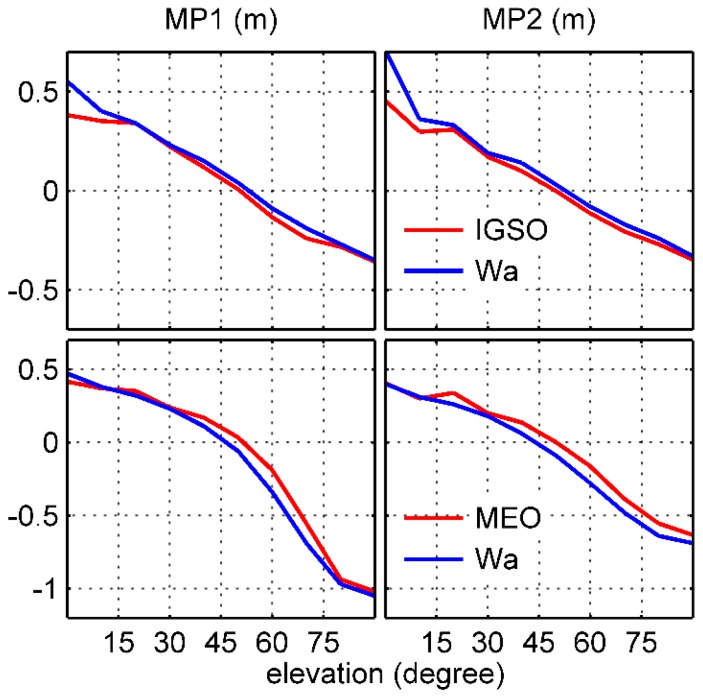
Comparison between the final derived model values and those of Wanninger and Beer.

Table 2 and Figure 11 show the comparison with Wanninger and Beer’s model values. There is a good fit between the lines even if the elevation angle falls below 40°. The RMS of the differences is less than 10 cm. Specifically, the differences for IGSO at zero elevation seem relatively larger. This can be attributed to the contamination of the relative larger noise at low elevation. Also, Wanninger and Beer’s MEO model values are slightly smaller than those of the derived model, which results in a grid of MEO with relatively larger values after application of Wanninger and Beer’s MEO model in Figure 9. This also explains the remaining trend components in IGSO-MEO panels. But these differences seem only marginal when considering the magnitude of the systematic variations.

**Figure 12:**
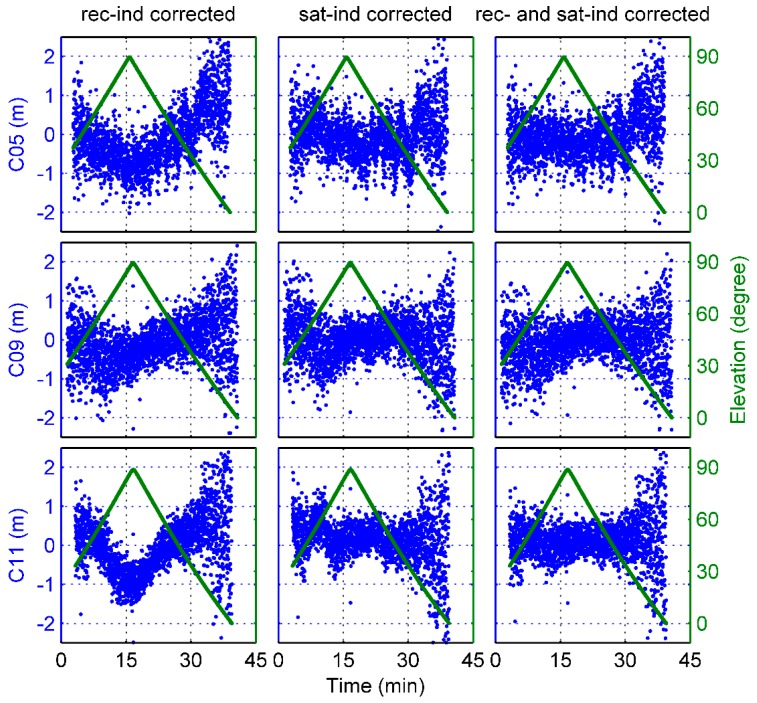
An example of MP1 variations after application of the GNOS-receiver-induced (rec-ind) and BDS satellite-induced (sat-ind) bias corrections for different BDS satellite types.

**Figure 13:**
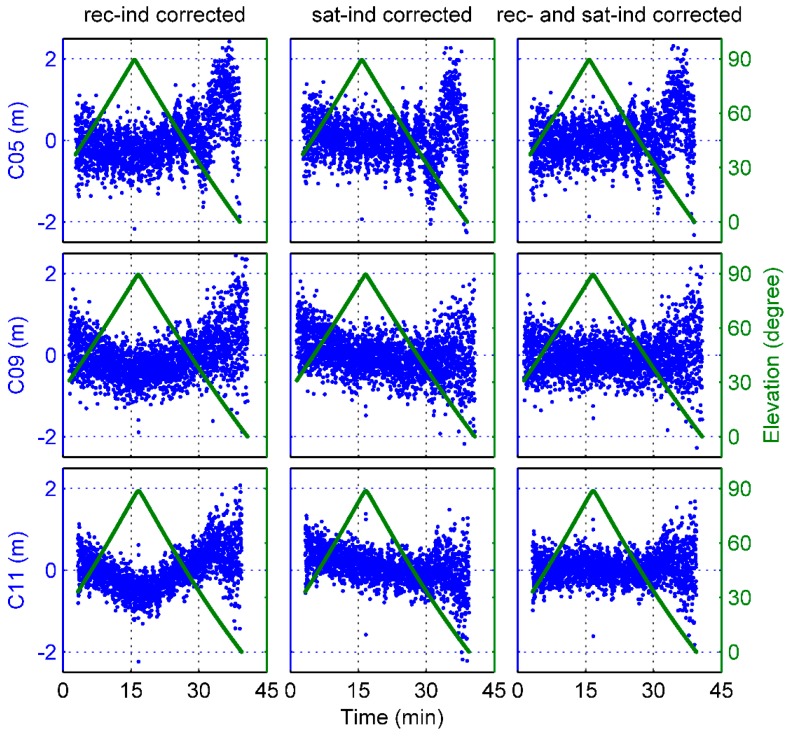
An example of MP2 variations after application of the GNOS-receiver-induced (rec-ind) and BDS satellite-induced (sat-ind) bias corrections for different BDS satellite types.

To verify the correctness of the derived models for the BDS-satellite-induced biases and the static GNOS-receiver-induced biases, some examples of MP combinations are shown. The same satellite arcs are used as those in Figure 4. Figure 12 and Figure 13 show that the MP time series regained the symmetry in elevation after application of the receiver-induced bias corrections for each of the 2 BDS frequencies. However, if only the satellite-induced bias corrections was used, the remaining receiver-induced biases was very evident in the MP variations, as shown in the Figures. When both model corrections were applied, these systematic biases disappeared. Regardless of the effect of the periodic signal mentioned above, the MP time series already shows the typical elevation-dependent variations, which are smallest for high elevations and largest for the ascending or descending satellites.

### 3.3. The Effect on Single-Frequency BDS Orbit Determination of Fengyun-3C

In previous sections, we analyzed the BDS MP effects based on the Fengyun-3C onboard data set, and found that the code measurements are not only subject to the BDS-satellite-induced and elevation-dependent systematic biases, but also suffer from the Fengyun-3C local near-field MP effects. Based on the analysis of the MP variations, we carefully modeled the BDS-satellite-induced biases of the corresponding group and the static GNOS-receiver-induced MP pattern. For dual-frequency precision orbit determination, the solution quality is mainly dependent on the carrier-phase observations but not the code measurement; thus the code biases can hardly affect the final orbit determination precision. However, code measurements can play an important role in single-frequency orbit determination, because in that case the graphic combination is used as a basic observation model, which enables the elimination of ionospheric delay but depends on code measurement precision [15,16]. Based on a single-frequency GPS orbit determination, the 3D RMS of the gravity recovery and climate experiment (GRACE) is better than 0.2 m, and the Chinese HY-2A and ZY-3 satellites can reach the same level [17,18]. In this section, to analyze the effect on the single-frequency BDS orbit determination of the Fengyun-3C satellite, we determined precise orbits with the onboard BDS data, and generated the orbit determination solutions with and without the model corrections using the positioning and navigation data analyst (PANDA) software package [19,20] developed at the GNSS Research Center of Wuhan University.

Because the Fengyun-3C satellite does not carry a satellite laser reflector, which is generally used to independently validate the accuracy of precise orbit products, we compared the results to the orbits using onboard dual-frequency GPS data provided by Zhao et al. [5]. The 3D RMS of overlapping orbit differences is better than 3 cm for a dual-frequency GPS solution. Therefore, the dual-frequency GPS orbit results can be seen as reference orbits to judge the accuracy of both the single-frequency solutions. Also, we adopted the BDS precise orbit and 30-s clock products provided by Wuhan University for both BDS solutions. The qualities of BDS products were evaluated by Guo et al. [21] and Guo et al. [22]. The detailed strategy used for orbit determination solutions is summarized in Table 3.

**Figure 14:**
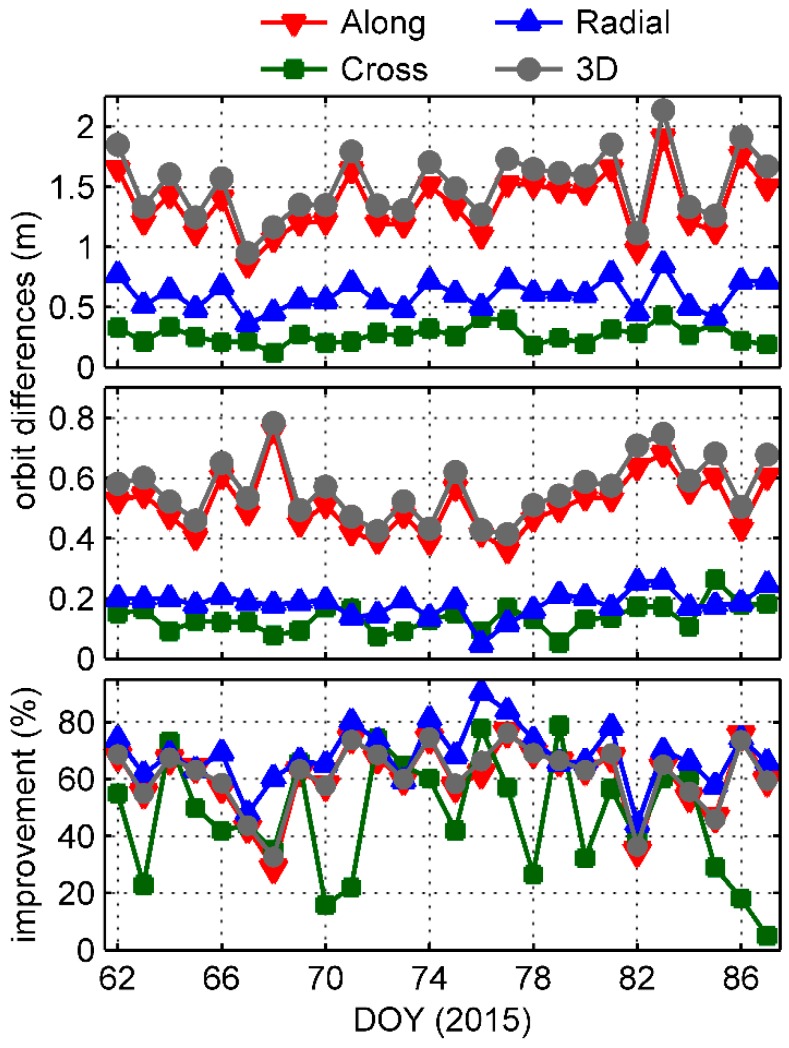
Daily RMS of orbit differences between the dual-frequency GPS solution and the single-frequency BDS solutions before (**top**) and after (**middle**) application of the receiver- and satellite-induced code bias model corrections and the improvements (**bottom**) when using corrections.

Figure 14 shows the daily RMSs of orbit differences between GPS and BDS solutions before and after application of the receiver- and satellite-induced code bias model corrections in along-track, cross-track, radial, and 3D respectively, and the improvement percentages. After applying the corrections, the RMS values are clearly improved in each direction. Particularly in the along-track component, the mean RMS is reduced from 135.3 to 51.2 cm with an improvement of 62.1%. The mean improvements in cross-track and radial components are 49.7% and 69.5% respectively. Among the improvements of the 3D mean RMS, which decrease from 150.6 to 56.3 cm, 78.8% comes from the along-track component, and 2.0% and 19.2% from the cross-track and radial respectively. The comparative analysis shows that the BDS-satellite- and GNOS-receiver-induced code biases seriously degrade the precision of Fengyun-3C orbits. However, the precision of the single-frequency BDS orbit determination is still not in competition with that of GPS. This is mainly because the available BDS data quantity is obviously less. Considering the Asia-Pacific regional service of BDS in 2015, only 3 MEO satellites were visible outside the Asia-Pacific region, and the number of GNOS positioning antenna tracking satellites is less than 4 at approximately two-thirds of the epochs, which typically contributes to poor positioning accuracy. In addition, the relatively lower precision of BDS products is also an inevitable problem for orbit determination.

## 4. Conclusions

Besides BDS-satellite-induced biases, onboard BDS code measurements from the Fengyun-3C GNOS receiver are subject to strong local MP effects, which result in a static pattern depending on frequency and the line-of-sight direction relative to the receiver antenna. There is a decreasing trend of MP in the static pattern from the signal ascending direction to the signal descending direction; its variation is larger than 1 m. Thus, removal of the local MP effects is necessary if the Fengyun-3C measurements are used to analyze the characteristics of BDS signals. We identified that there is no dependence of the local static pattern on BDS satellite type. Therefore, we estimated an elevation- and azimuth-dependent grid model for the static pattern together with the groups of the linear piece-wise parameters of the BDS-satellite-induced biases for each of the 2 BDS frequencies. The derived linear model values for BDS IGSO and MEO code biases agreed with those of Wanninger and Beer’s model even if the elevation angle falls below 40°. Also, the linear model parameter values of BDS GEO satellites from 0° to 90° can be obtained at the same time, which is nearly impossible using ground-fixed measurements due to small movements of the GEO satellites relative to the ground receiver. Compared with other GEO satellite B1 model values, a different behavior really existed in C05 and a new group for C05 B1 was considered. Also, the derived B2 linear model values for the GEO and IGSO groups were so similar that we suggest they should be placed in the same category. Apart from an increasing deviation when the elevation angle was above 60°, the difference between groups in the same frequency was less than 5 cm except for the C01 to C04 B1 group.

To analyze the effect of these code systematic biases on the orbit determination of the Fengyun-3C satellite, we generated 2 single-frequency BDS orbit determination solutions using the nearly one-month onboard data set. There was a noticeable improvement in each direction when we applied the GNOS-receiver- and BDS-satellite-induced code bias model corrections. In particular, the mean RMS in the along-track component decreased from 135.3 to 51.2 cm, which accounted for 78.8% of the 3D improvement from 150.6 to 56.3 cm. The result shows a substantial contribution from these corrections. However, due to BDS data quantity being less available and the relatively lower precision of BDS products, the single-frequency BDS orbit determination precision is still not in competition with that of GPS.

## Figures and Tables

**Table 1:** Seven groups of the final derived elevation-dependent correction node values for BDS-satellite-induced biases.

Elevation (°)	Linear Model Node Values (m)						
	GEO			IGSO		MEO	
	B1 (C01–C04)	B1 (C05)	B2	B1	B2	B1	B2
0	−0.14	−0.43	−0.46	−0.38	−0.45	−0.42	−0.41
10	−0.25	−0.37	−0.27	−0.35	−0.30	−0.37	−0.30
20	−0.27	−0.31	−0.26	−0.34	−0.31	−0.35	−0.34
30	−0.18	−0.17	−0.14	−0.22	−0.17	−0.24	−0.20
40	−0.09	−0.14	−0.09	−0.12	−0.10	−0.17	−0.14
50	−0.05	−0.01	0.01	−0.01	0.00	−0.03	−0.00
60	0.03	0.17	0.12	0.13	0.11	0.19	0.16
70	0.12	0.32	0.20	0.24	0.21	0.56	0.39
80	0.12	0.45	0.25	0.28	0.27	0.94	0.56
90	0.22	0.69	0.31	0.36	0.35	1.02	0.63

**Table 2:** The differences between the derived values and Wanninger and Beer’s model.

Elevation (°)	Differences with Wanninger and Beer’s Model (m)			
	IGSO		MEO	
	B1-Wa	B2-Wa	B1-Wa	B2-Wa
0	−0.17	0.26	0.05	−0.01
10	−0.05	0.06	0.01	0.01
20	0.00	0.02	−0.03	−0.08
30	0.01	0.02	−0.01	−0.02
40	0.03	0.04	−0.06	−0.08
50	0.03	0.03	−0.09	−0.09
60	0.04	0.03	−0.15	−0.12
70	0.05	0.04	−0.14	−0.09
80	0.01	0.03	−0.03	−0.08
90	0.01	0.02	−0.03	−0.06
AVG	0.00	0.06	−0.05	−0.06
RMS	0.06	0.09	0.08	0.07

**Table 3:** Summary of the dynamical and measurement models employed for the Fengyun-3C orbit determination solutions based on onboard BDS data.

Items	Description
Gravity model	EIGEN-06C, up to degree and order 120 for static field and 50 for time-varying gravity
Precession and nutation	IERS 2010 [23]
Earth orientation	IERS C-04 [24]
Solid Earth tide and pole tide	IERS 2010 [23]
Ocean tide	FES2004 30 × 30 [25]
Ocean pole tides	Desai [26]
N-body perturbation	JPL DE405
Relativity	IERS 2010 [23]
Solar radiation pressure	Box-wing model
Atmosphere drag	DTM2013 [27], piece-wise drag coefficients estimated every 360 min
Attitude	Nominal
Fengyun-3C PCO (X/Y/Z: m) & PCV	−1.2750/0.2820/−0.9837, PCV not considered
BDS PCO & PCV	model from Guo et al. [21]
Empirical forces	piece-wise periodical terms in along- and cross-track direction estimated every 360 min
BDS observation type	B1
Observation interval	30 s
BDS orbit and clock	Wuhan University precise products (30-s clock)
ionosphere delay	Graphic combination
orbit determination arc length	30 h
Elevation cutoff	0°
Fengyun-3C initial state	Position and velocity at initial epoch
Receiver clock	One per epoch as process noise
Ambiguities	One per pass
